# An Egyptian child with erythromelalgia responding to a new line of treatment: a case report and review of the literature

**DOI:** 10.1186/1752-1947-8-69

**Published:** 2014-02-25

**Authors:** Samir M Al-Minshawy, Abdel-Azeem M El-Mazary

**Affiliations:** 1 Department of Pediatrics, Minia University, Postcode 61111 Minia city, Egypt

**Keywords:** Cetirizine hydrochloride, Egyptian children, Erythromelalgia

## Abstract

**Introduction:**

Erythromelalgia is a rare clinical syndrome characterized by episodic erythema, warmth and intense burning pain, which commonly involves the extremities. For those affected, this disorder may lead to significant long-term morbidity. Unfortunately, to date, no definitive therapy is available. This case report describes an Egyptian child with primary erythromelalgia that manifested at an early age and showed partial response to therapy with cetirizine hydrochloride. This anecdotal case report may have a diagnostic value for clinicians who have not seen this disorder.

**Case presentation:**

A 34-month-old previously healthy right-handed Hamitic boy without any significant past medical history presented at the age of 2 years with episodic bilateral pain in his feet. His mother reported associated warmth and erythema localized to his feet that never extended beyond his ankle joints. This pain is triggered by exertion and/or warm temperature exposure and is relieved by cooling measures. The diagnosis of erythromelalgia was made based on the patient’s medical history and a thorough physical examination during the episodes. No evidence of local or systemic infection was present. Other causes for the symptoms were excluded by a negative extensive diagnostic work-up. Our patient did not respond to ibuprofen (15mg/kg/dose) three times a day but partial improvement with the oral non-sedating antihistaminic cetirizine hydrochloride (2.5mg/kg/once daily) was observed. When the child stopped cetirizine hydrochloride for 1 month as a test, the symptoms became aggravated and were relieved when cetirizine therapy was restarted. Cetirizine hydrochloride had not previously been reported to have this effect in children with erythromelalgia.

**Conclusions:**

Erythromelalgia is a clinical syndrome of which the etiology, diagnosis and management are controversial. We describe a case of a 34-month-old Egyptian child with primary erythromelalgia that manifested at an early age. We believe that this is the first Egyptian case report of this kind in the literature. Partial response of this patient to cetirizine hydrochloride may grant us a new clue to understanding this mysterious condition.

## Introduction

Erythromelalgia (EM) is a rare disorder in children characterized by episodic erythema, warming and burning pain, which commonly involves the extremities [1]. EM can be primary (which may be sporadic or familial) or secondary to other causes including but not limited to autoimmune disorders, myeloproliferative and/or neuropathic conditions [2] (Table 1).

**Table 1:** **Reported causes of secondary erythromelalgia**[2]


**Myeloproliferative diseases and blood disorders**	**Drugs**
Essential thrombocythemia	Cyclosporine
Polycythemia vera	Verapamil
Myelodysplastic syndrome	Nicardipine
Pernicious anemia	Nifedipine
Thrombotic and immunologic thrombocytopenic purpuras	Norephedrine
	Bromocriptine and pergolide
**Infectious diseases**	**Connective tissue diseases**
Human immunodeficiency virus	Systemic lupus erythematosus
Hepatitis B vaccine	Vasculitis
Influenza vaccine	**Neoplastic**
Infectious mononucleosis	Paraneoplastic syndrome
Pox virus	Astrocytoma
**Neuropathic**	Malignant thymoma
Diabetic neuropathy	**Others**
Peripheral neuropathies	Mushroom ingestion
Neurofibromatosis	Mercury poisoning
Riley–day syndrome	
Multiple sclerosis	

Symptoms are triggered by physical exertion and/or a warm environment and can be relieved by cooling. Episodes may last from minutes to hours. Early recognition of EM is important but difficult due to the rare nature of the disorder [3].

## Case presentation

**Figure 1:**
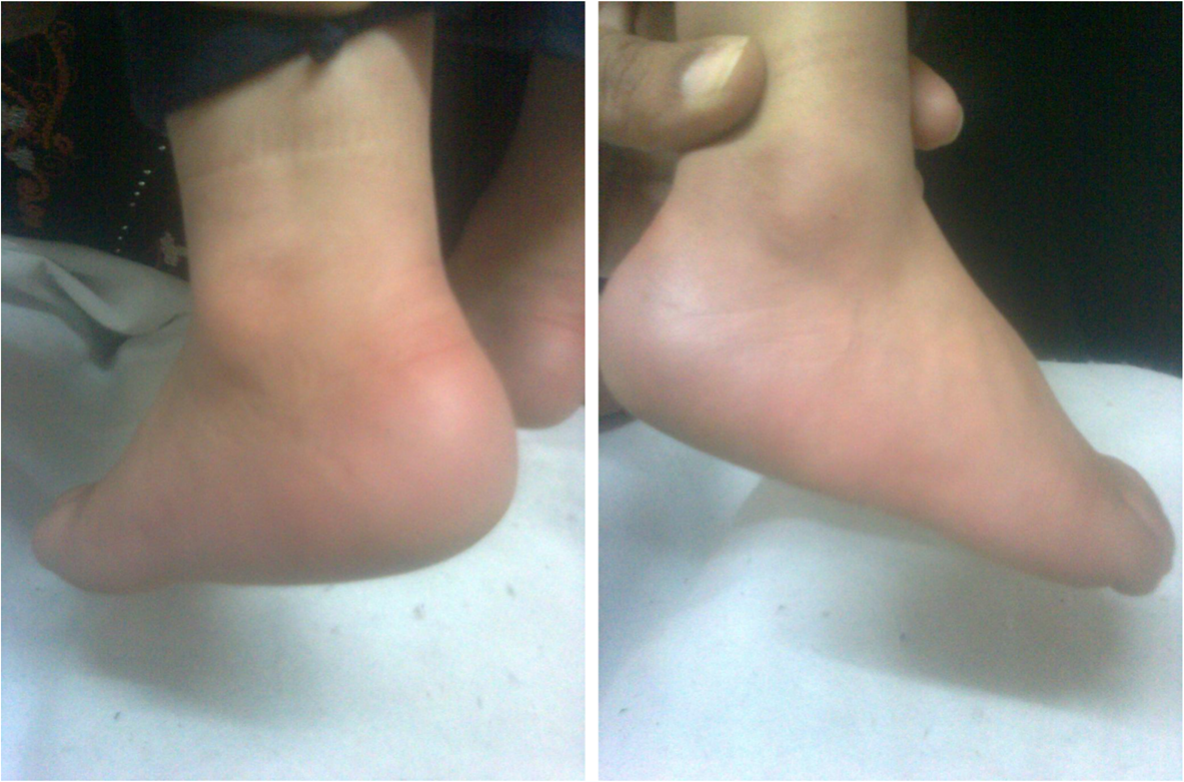
Photography for our case during an episode.

Our patient was a 34-month-old right-handed Hamitic boy who presented with insidious intermittent attacks of bilateral intense pain, warmth and flushing of feet, each lasting minutes to hours. The age of onset for these symptoms was 2 years. His symptoms had the tendency to be symmetrical, localized to his feet and never extended proximally beyond his ankle joints; they were precipitated and worsened with exercise and/or warm temperature exposure such as covering his legs with blankets and were abated by cooling measures like cold water. There was no history of similar conditions in his family or drug intake before the precipitation of the attacks. He had no history of previous blood transfusion. He looks well, with no manifestations of acute illness. Physical examinations during multiple visits revealed: normal vital signs; no pallor, jaundice or cyanosis were present; no organomegaly or lymphadenopathies were present; only both his feet appeared red in color (Figure 1) and warm. Extensive investigations were done for exclusion of other diseases causing pain and/or flushing of both lower limbs as well as for exclusion of secondary EM.

In this child, the investigations revealed normal complete blood count (CBC) with differential, normal serum immunoglobulin E (IgE) titre (11IU/mL), normal levels of serum cholesterol (146mg/dL) and triglycerides (49mg/dL), normal levels of serum urea (16mg/dL) and serum creatinine (0.7mg/dL), normal liver enzymes (alanine aminotransferase 22U/L and aspartate aminotransferase 34U/L), normal serum uric acid (3.7mg/dL), negative antistreptolysin O titre, and normal urine analysis and stool analysis. The fasting and 2 hours post-prandial blood glucose levels were 89 and 122mg/dL respectively.

In addition, the results of an X-ray of the bones in both his feet and legs and a Doppler of the arteries of both his lower limbs were normal, normal nerve conduction velocities of both peroneal nerves and normal bone marrow biopsy were present. A pelvic and abdominal sonography and brain computed tomography (CT) were done and all were normal.

**Figure 2:**
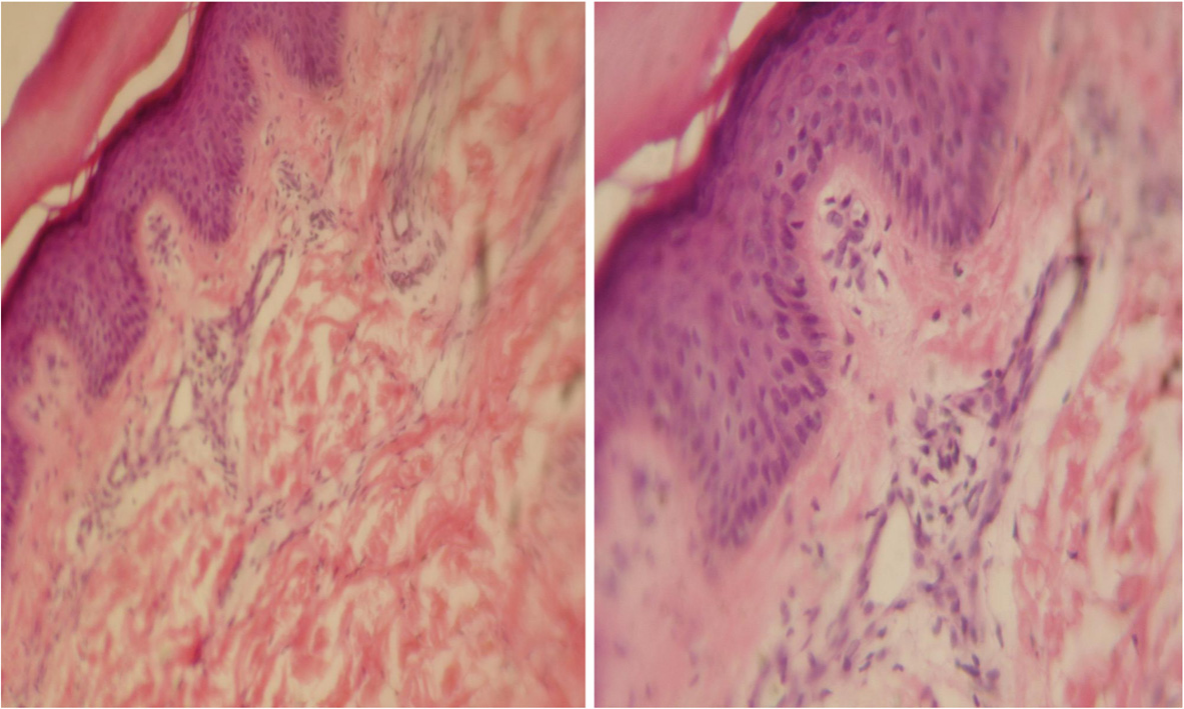
Skin biopsy of the case.

A skin biopsy was performed, showing nonspecific changes consistent with the diagnosis of primary EM (Figure 2) in the form of numerous telangiectatic blood vessels in the capillary dermis associated with sparse perivascular mononuclear cell infiltrate and some vessels showed swelling of the endothelial lining. The intimal thickening and thrombi seen in secondary EM were lacking.

He received ibuprofen (15mg/kg/dose three times a day) for 2 to 3 weeks but no relief of his symptoms was observed, but he did report a partial response to cetirizine hydrochloride (2.5mg/kg/once daily). When the child stopped cetirizine hydrochloride for 1 month as a test, his symptoms became aggravated but were relieved when cetirizine therapy was restarted; the frequency and severity of the attacks were reduced. His mother was advised that he should avoid all conditions that exacerbated his symptoms and that she should expose his lower limbs to cold air (fan) during an attack and avoid his exposure to cold or ice water to avoid ischemia of both his lower limbs. She was advised on the benefit of routine follow-up evaluation in the pediatric clinic for follow-up by CBC and, if necessary, any other investigations needed. Finally, the diagnosis of primary EM was made based on the clinical history and examination.

## Discussion

In 1878, Mitchell [4] described and named ‘erythromelalgia’; the name ‘erythromelalgia’ reflected the characteristic findings of redness (erythros) and pain (algos) involving the extremities (melos). Unfortunately, there is at present no confirmatory diagnostic test and the diagnosis is based on taking a careful history and physical examination during the episodes. With the advent of digital photography, photographs can be very helpful to document the events of erythema. Thermography can reveal the increased skin temperature in the affected area, but this is not necessary to establish the diagnosis [2,3].

The incidence and prevalence of EM are difficult to calculate due to the patient’s failure to recognize the condition when the symptoms are mild, and physicians may fail to make the diagnosis because this is a rare and relatively unknown disorder. The proposed incidence is 2.5 to 3.3 per million per year [5], with a prevalence of 18 to 20 per million in the Norwegian population [6]. A recent retrospective study in Sweden reported an incidence of 0.36 cases per 100,000 population [7]. The incidence and prevalence of EM in Egypt is unknown and it should be noted that this case may be the first case in Egypt reported.

Adult-onset EM has a wide range of age distribution, with most cases occurring in the fifth and sixth decades; in early reports, the median age of onset for early-onset EM was 10 years [5].

After a 37-year analysis of 32 pediatric cases at the Mayo Clinic, Minnesota, researchers concluded that the majority of cases were not inherited and that progress of the disease is variable. No safe or reliable treatment has been established; EM in the pediatric population is associated with substantial morbidity and, sometimes, death [8].

In a Norwegian case series [7], the age range in the primary group (early onset and idiopathic adult onset disease) was 7 to 76 years, and the age range in the secondary group (secondary EM) was 18 to 81 years. In a retrospective review of 168 patients with EM examined at the Mayo Clinic between 1970 and 1994, the median age was 60 years and the age range was 5 to 91 years [5,8].

The early age of our child is considered one of the earliest ages for presentation for primary (or idiopathic) EM and this is consistent with the few studies that have reported onset of EM at the age of 3 years or less in some families [9-11].

The Mayo Clinic series showed a male-to-female ratio of 1:3, whereas it was 3:2 in a review of 60 cases of EM due to myeloproliferative disorders [5,7,8].

As there is a relatively well-documented association between EM and myeloproliferative disorders and systemic mastocytosis, an initial CBC with a differential count as well as bone marrow examination were done which revealed normal values. A normal CBC excluded other hematological disorders including pernicious anemia, essential thrombocythemia, polycythemia vera, myelodysplastic syndrome, and thrombotic and immunologic thrombocytopenic purpuras [1,3]. Hemoglobin electrophoresis was done for the exclusion of chronic hemolytic anemias, especially sickle cell anemia, which may elucidate the symptomatology of the child when no abnormality can be detected.

Several authors [5,7] have reported that the onset of symptoms of EM may precede detectable myeloproliferative diseases by several years. So our patient was advised on the benefit of routine follow-up evaluation. EM may be secondary to connective tissue disorders [2], such as rheumatoid arthritis, systemic lupus erythematosus, Sjögren’s syndrome and vasculitis, but the clinical presentation and laboratory investigations excluded them because the results of the autoimmune panel were normal. This autoimmune panel included: normal CBC and erythrocyte sedimentation rate, negative rheumatoid factor, and antinuclear, anti-Smith and anti-deoxyribonucleic acid (DNA) antibodies.

Some drugs (for example bromocriptine), mushroom ingestion and mercury poisoning [2,12] were reported to cause secondary EM and those were excluded by history and clinical examination.

In our case, there was no X-ray evidence of calcified arteries in his lower limbs. In addition, he had normal blood pressure, normal values of serum cholesterol and triglycerides levels, and normal blood flow of both lower limbs through the Doppler which ruled out EM-like syndromes such as Raynaud’s syndrome and Buerger’s disease.

Neuropathies (including diabetic neuropathies), multiple sclerosis and spinal cord disease are some of the neurological disorders associated with EM [2]; these were excluded through normal urine analysis, fasting and 2 hours post-prandial blood glucose levels, and normal nerve conduction velocity of both peroneal nerves. The results of a pelvic and abdominal sonography and brain CT, which were done to exclude oncologic causes especially astrocytoma, were normal.

The exact pathological mechanism responsible for this disorder is unknown, but several theories have been proposed to explain its pathophysiology including vasculopathy and/or neuropathy hypotheses. According to the microvascular arteriovenous (AV) hypothesis [13,14], symptoms are caused by tissue hypoxia induced by impaired distribution of skin microvascular blood flow with increased thermoregulatory flow through AV shunts and an inadequate perfusion. Work done by Mork and colleagues [15] supports this hypothesis of increased thermoregulatory flow through AV shunts during attacks in primary EM, whereas Orstavik and colleagues suggested that EM may be due to hypersensitivity of C-fibers [16].

A prospective study done by Davis *et al.*[17] suggested that EM is associated with a neuropathy, primarily small-fiber, and a vasculopathy with intermittent increased blood flow and AV shunting. There may be increased local cellular metabolism. However, it is unclear which one is the initiating event or primary abnormality.

Kim *et al*. [18] reported mutations in the *SCN9A* gene, which encodes the Na_v_1.7 sodium channel, in patients with primary EM. Cummins *et al*. [19] demonstrated that these mutations in the Na_v_1.7 channel produce a hyperpolarizing shift in activation and slow deactivation causing the sodium channels to remain open for extended periods of time.

Dib-Hajj *et al*. [20,21] demonstrated another F1449V mutation in the Na_v_1.7 channel, which also reduces the firing threshold and produces abnormal repetitive firing in sensory neurons in primary EM. Nearly a dozen mutations in Na_v_1.7 have been identified. These mutations have been found to be a cause of familial EM.

Drenth and Waxman [22], Dib-Hajj *et al*. [23] and Min-Tzu and colleagues [24] reported that mutations in the human *SCN9A* gene, encoding the α–subunit of the voltage-gated sodium channel, Na_v_1.7, were found to be responsible for primary EM. Three missense mutations of the *SCN9A* gene have recently been identified in Taiwanese patients including a familial (I136V) and two sporadic mutations (I848T, V1316A). V1316A is a novel mutation and has not been characterized yet. Topologically, I136V is located in the DI/S1 segment and both I848T and V1316A are located in S4-S5 linker region of DII and DIII domains, respectively.

Overall, these changes reflect the hyperexcitability of peripheral sensory and sympathetic neurons, which contributes to symptom production in primary EM.

A skin biopsy was done for our child and revealed nonspecific changes as mentioned before; this is consistent with many studies that revealed the same changes [14,15,25].

A universally effective treatment for primary (or idiopathic) EM is still unknown as shown in Table 2. The mainstay of therapy is support and avoidance of trigger factors. Local measures include cooling or elevating the extremity to effectively attenuate or relieve symptoms. Patients should also be counseled about the use of safe cooling options such as fans and air conditioning rather than cold water itself, as ice or immersing an extremity into an icy water bath can lead to skin necrosis and ulceration [5,8].

**Table 2:** **Response to earlier medications and other treatments in 32 patients presenting to Mayo Clinic with erythromelalgia**[8]

**Drugs used**	**No. of patients, response**			
	**n**	**Very helpful**	**Somewhat helpful**	**Not helpful**
Aspirin	14	1	2	11
NSAIDs *(ibuprofen, indomethacin, naproxen)*	14	1	0	13
Antidepressants *(amitriptyline hydrochloride, venlafaxine, cyproheptadine hydrochloride)*	10	2	2	6
Antihistamines *(diphenhydramine, cetirizine hydrochloride, cimetidine)*	8	0	0	8
Vasodilators *(nitroprusside, nifedipine, diltiazem)*	7	1	1	5
Β-blockers *(propranolol, atenolol, nadolol)*	6	0	1	5
Narcotics *(codeine, morphine, fentanyl)*	6	0	4	2
Gabapentin	6	2	0	4
Parenteral corticosteroids *(oral, intramuscular, intravenous)*	6	0	1	5
Topical corticosteroids	5	0	0	5
Physical methods *(biofeedback, intrathecal pump, TENS unit)*	4	1	1	2
Other anticonvulsants *(carbamazepine, phenytoin)*	3	0	1	2
Sympathectomy	3	0	1	2
Acetaminophen	3	0	1	2
Other medications* (doxazosin mesylate, capsaicin, ergotamine tartrate, mexiletine, clonidine, tetracycline, homeopathic)	7	0	0	7

*NSAIDs,* nonsteroidal anti-inflammatory drugs; *TENS, transcutaneous electrical nerve stimulation.*

*Each drug given to one patient and all of them gave a “not helpful” response.

Treatment with medications such as propranolol, gabapentin, tricyclic antidepressants, sodium nitroprusside, calcium channel blockers and intravenous lidocaine and oral mexiletine had some symptomatic benefits in a few cases and mainly in adults not in children [26-31].

Medications that affect voltage-gated sodium channels show promise, although prostacyclin may provide some benefit and some patients achieved relief with gabapentin or high-dose magnesium [32-34]. Some rare patients with EM may respond well to treatment with carbamazepine especially those with the Na_v_1.7channel mutations [35].

Recent studies have indicated that it is possible to predict the response of patients with EM to treatment with sodium channel blockers on the basis of atomic-level structural modeling [36] raising the possibility that, in the future, it may be possible to genotype patients with EM, and prospectively predict the response to various drugs via pharmacogenomics. We could not perform a genetic study in our case because this was expensive; we depended on the clear history and the clinical picture for the diagnosis of our case. Lastly, genetic study may be more valuable in studies comparing different modalities of therapy.

Antihistamines are often overlooked in the treatment of EM, but these drugs have potent vascular effects and should be considered in difficult cases [37]. Published reports described two remissions with cyproheptadine and three cases with marked improvement using pizotifen, which are antihistamines with proven serotonin antagonist effects at 5-HT2 receptors. In its June 2006 newsletter, The Erythromelalgia Association (TEA) reported a remission that has lasted 10 years with a low dose of cyproheptadine [37,38].

The TEA survey indicates that approximately 40% of users of antihistamines obtain modest improvement in their EM, whereas 60% do not obtain improvement. One TEA member reported marked improvement with variable use of desloratadine, chlorpheniramine, and diphenhydramine [37].

Regarding the non-sedating antihistaminic, cetirizine hydrochloride, no improvement was observed in some previous reports [2,8,37] which is in contradiction to our case. This may lead us to think about a new theory of chronic long-standing local allergic reaction which enlightens response to cetirizine in our case in spite of a normal serum IgE titre on the basis of atomic-level structural modeling.

Secondary EM successfully treated with intravenous immunoglobulin plus treatment of the cause in a female patient with seronegative polyarthritis has also been reported [39].

Ulceration, necrosis, and gangrene of affected extremities are possible. Digital necrosis or skin ulceration with secondary infection can lead to amputation. At least one patient had near-fatal hypothermia related to the constant cooling required to control symptoms [5,8,37].

## Conclusions

EM is a rare clinical syndrome of which the etiology, diagnosis and management are controversial. We describe a case of a 34-month-old Egyptian child with primary EM that manifested at an early age. We believe that it is one of the rare cases with early onset of presentation and we believe it is the first Egyptian case report of this kind in the literature. Partial response of this case to cetirizine hydrochloride may grant us a new clue to understanding this mysterious condition.

## Consent

Written informed consent was obtained from the child’s father for publication of this case report and any accompanying images. A copy of the written consent is available for review by the Editor-in-Chief of this journal.

## Abbreviations

AV: Arteriovenous; CBC: Complete blood count; CT: Computed tomography; EM: Erythromelalgia; IgE: Immunoglobulin E; TEA: The Erythromelalgia Association.

## Competing interests

The authors declare they have no competing interests.

## Authors’ contributions

SA managed the patient, gathered the data, searched and reviewed the literature. AE searched and reviewed the literature and critically shared in the design and finishing the manuscript. Both authors read and approved the final manuscript.
